# Hydrophobic Covalent Organic Frameworks as a Platform for Instantaneous and High‐Capacity Immobilization of *Diutina* (*Candida*) *rugosa* Lipase: A Simple Strategy to Boost Enzyme Activity

**DOI:** 10.1002/cplu.70284

**Published:** 2026-11-04

**Authors:** Leticia Dobler, Mirian Lima de Almeida Lippi, Luiz Octávio Mateus Nogueira, Davi Forastieri Tostes, Alexandre da Silva França, Fábio Junior Ferreira da Silva Henrique, Leandro Sodré de Abreu, Gabriel Almeida Mendonça da Silva, Cesar Aguzzoli, Sidnei Moura e Silva, Rodrigo O. M. A. de Souza, Rodrigo Volcan Almeida, Pierre Mothé Esteves, Raoni Schroeder Borges Gonçalves

**Affiliations:** ^1^ Instituto de Química Universidade Federal do Rio de Janeiro Rio de Janeiro Brazil; ^2^ Programa de Pós‐Graduação em Ciência e Engenharia de Materiais (PPGMAT) Universidade de Caxias do Sul (UCS) Caxias do Sul Rio Grande do Sul (RS) Brazil; ^3^ Departamento de Tecnologia LBIOP – Laboratório de Biotecnologia de Produtos Naturais e Sintéticos Instituto de Biotecnologia Universidade de Caxias do Sul Caxias do Sul Brazil

**Keywords:** biocatalysis, COF, enzyme, heterogeneous catalysis, nanoporous material

## Abstract

Covalent Organic Frameworks (COFs) are emerging as promising platforms for enzyme immobilization; however, the impact of their structural and chemical properties on adsorption and enzymatic activity remains underexplored. Here, *Diutina rugosa* lipase (DrL) was immobilized on four COFs (RIO‐12, RIO‐13, RIO‐54, and RIO‐55) synthesized from simple, commercially available precursors. The effects of COF chemical composition and textural properties on adsorption dynamics and catalytic performance were evaluated. Immobilization occurred almost instantaneously, reaching completion in less than 10 s regardless of the COF used, with loading efficiencies of 99.8%–99.9%, protein binding capacities of approximately 44 mg g^−1^ of support, and enzyme leaching below 0.3% per washing cycle for all composites. Our results show that fully organic COF surfaces, rich in aromatic and nitrogen‐containing functional groups, exhibit a strong affinity for lipases, enabling rapid immobilization primarily driven by hydrophobic interactions, whereas pore size and surface area play a secondary role. In contrast, enzymatic activity was strongly influenced by surface area, likely through a positive partition effect that increases the local substrate concentration near the enzyme. Notably, RIO‐12 exhibited a remarkable enhancement in activity, achieving a specific activity 14.8‐fold higher than that of the free enzyme (408.5 vs. 27.7 U mg^−1^).

## Graphical abstract

High‐surface‐area hydrophobic covalent organic frameworks enable rapid enzyme immobilization with high loading efficiency, enhancing the specific enzymatic activity by up to 14.8‐fold. The synergistic effects of surface area, hydrophobicity, and framework structure provide an efficient platform for enzyme immobilization and catalytic applications.

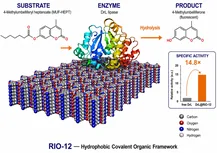

## 1 Introduction

Among the various classes of enzymes with biotechnological relevance, lipases (triacylglycerol acylhydrolases, EC 3.1.1.3) stand out because of their remarkable catalytic versatility. They catalyze a wide range of reactions, including hydrolysis, esterification, interesterification, transesterification, alcoholysis, acidolysis, and aminolysis [1], making them valuable biocatalysts for applications in the food, pharmaceutical, chemical, and biofuel industries. Despite these advantages, the industrial use of free lipases is often limited by poor operational stability, difficult recovery, and limited reusability. Immobilization onto solid supports has therefore become an effective strategy to overcome these drawbacks by improving enzyme stability, facilitating catalyst recycling, and, in many cases, enhancing catalytic performance. The success of this strategy depends not only on the immobilization protocol but also on the structural and chemical characteristics of the support, including its surface chemistry, hydrophobicity, pore architecture, and accessible surface area [2, 3].

Among the wide variety of porous materials investigated for enzyme immobilization, Covalent Organic Frameworks (COFs) have recently emerged as particularly attractive platforms. COFs are crystalline porous materials constructed entirely from organic building blocks through strong covalent bonds, combining high surface areas, tunable pore structures, excellent chemical stability, and exceptional synthetic versatility [4]. These features enable the rational design of tailored microenvironments capable of modulating enzyme adsorption, stability, and catalytic behavior. Consequently, COFs have attracted increasing attention as next‐generation enzyme supports for heterogeneous biocatalysis [5, 6, 7, 8, 9].

Several immobilization strategies have already been explored using COFs. Functionalized frameworks have been employed to enhance enzyme‐support interactions through both physical adsorption and covalent attachment [10, 11, 12, 13, 14, 15, 16]. For example, Sun et al. [10]. demonstrated that the incorporation of specific functional groups into COF structures significantly influenced the activity of immobilized Amano lipase PS, highlighting the importance of the local chemical environment. In our previous work, an imine‐linked COF (PPF‐2) containing surface amino or aldehyde groups enabled the covalent immobilization of *Candida antarctica* lipase B, producing highly active biocatalysts with excellent thermal stability and recyclability [11]. Alternative strategies have also been reported, including the in situ assembly of enzymes during COF formation [16], immobilization within hierarchical mesoporous COFs [12, 13, 14, 15], and the development of scalable, environmentally friendly synthetic routes for large‐scale applications [16, 17]. Collectively, these studies demonstrate that COF‐enzyme composites have evolved beyond proof‐of‐concept materials and now represent a versatile platform for applications ranging from biosensing and asymmetric synthesis to biodiesel production and other industrially relevant processes [18].

Despite these important advances, fundamental questions regarding lipase [16, 17]immobilization on COFs remain unanswered. Most reported studies have focused on developing new COF‐enzyme composites or improving catalytic performance, whereas the mechanisms governing enzyme adsorption have received comparatively little attention. In particular, systematic investigations evaluating how COF surface chemistry and textural properties independently influence enzyme loading, adsorption kinetics, and catalytic activity are still lacking. To the best of our knowledge, neither adsorption kinetics nor adsorption isotherm studies have been systematically reported for lipase immobilization on COFs. Consequently, it remains unclear whether enzyme adsorption is primarily governed by surface chemistry, pore architecture, or accessible surface area. Moreover, the development of COFs synthesized from inexpensive, commercially available building blocks through straightforward and scalable procedures remains an important challenge for translating these materials into practical biocatalytic technologies.

To address these knowledge gaps, we investigated the immobilization of *Diutina rugosa* lipase (DrL) on four in‐house synthesized COFs (RIO‐12, RIO‐13, RIO‐54, and RIO‐55) prepared from simple, commercially available precursors. These materials were deliberately selected to provide systematic variations in surface area, pore size, and surface functional group density, enabling the independent evaluation of the influence of chemical composition and textural properties on enzyme immobilization. Adsorption kinetics and equilibrium isotherms were investigated to establish the relationship between COF structure and protein uptake, representing, to the best of our knowledge, the first systematic adsorption study of lipases on COFs. The catalytic performance of the resulting biocatalysts was subsequently evaluated through ester hydrolysis, allowing the relationship between enzyme adsorption and catalytic activity to be elucidated. Overall, this work establishes clear structure–performance relationships governing lipase immobilization on COFs and provides practical design principles for the rational development of low‐cost, scalable, and high‐performance COF‐based biocatalysts for industrial applications.

## 2 Materials and Methods

### 2.1 Synthetic Procedures

**Synthesis of 1,3,5‐triformylresorcinol:** 1,3,5‐triformylresorcinol was obtained through a previously described and adapted synthesis method [19]. Briefly: trifluoroacetic acid (90 mL) was added to a 500 mL round‐bottom flask and cooled to 0 °C in an ice bath, then hexamethylenetetramine (HMTA, 2 equivalents, 96 mmol, 13.46 g) was added. The solution was stirred until HMTA was completely dissolved, and resorcinol (1 equivalent, 48 mmol, 5.26 g) was then added slowly. The flask was then heated in a reflux system at 120 °C for 20 h. After this period, the solution turned yellow. 3 M hydrochloric acid acid (150 mL) was added, and the mixture was refluxed for an additional 4 h. The flask was removed from the heat and allowed to cool to room temperature, resulting in the formation of a precipitate. After cooling, the flask was placed in a −20 °C freezer for three more days to promote further precipitation. The solution was filtered and washed with ethanol, yielding the triformylresorcinol product (yellow solid) with a 70% yield.

**Synthesis of 1,3,5‐triformylphloroglucinol:** The synthesis of 1,3,5‐triformylphloroglucinol was followed as described by Chong et al. 2003 [20], under an argon atmosphere. Briefly, trifluoroacetic acid (90 mL) was added to a 500 mL round‐bottom flask and cooled to 0 °C in an ice bath. Hexamethylenetetramine (HMTA, 50 mmol, 7.56 g) was added, and the solution was stirred until the HMTA was completely dissolved. Phloroglucinol (20 mmol, 3.05 g) was then added, and the mixture was heated at 120 °C for 2 h and 30 min. After this period, the solution turned caramel color. 150 mL of 3 M HCl was added, and the mixture was heated at 100 °C for 1 h. The flask was cooled to room temperature, and the product was extracted with dichloromethane (350 mL). Magnesium sulfate was added to remove any residual water, and the solution was evaporated under vacuum to obtain the triformylphloroglucinol product (salmon solid) with a 20% yield.

**Synthesis of COFs RIO‐12 and RIO‐13:** COFs RIO‐12 and RIO‐13 have been reported in the literature and were obtained through solvothermal methods [19]. A glass pressure vessel was charged with the respective aldehyde (1 eq., 2 mmol). The top of the tube was closed with a rubber septum, and its atmosphere was changed by argon flow. After that, 13.6 mL of anhydrous dioxane, 1.2 mL of mesitylene and hydrazine hydrate 60% (1.5 eq., 3 mmol) were introduced into the tube through the rubber septum with a syringe, followed by addition of 4.6 mL of aqueous 6 M acetic acid. The tube was closed with a PTFE cap and heated at 120 °C for 72 h, yielding a solid at the bottom of the tube which was isolated by filtration and washed with anhydrous dioxane, dimethylformamide, methanol, and tetrahydrofuran (THF). The resulting powder was immersed in anhydrous THF for 72 h. After that, it was filtered off and dried under vacuum at room temperature (RT) overnight.

**Synthesis of RIO‐54 and RIO‐55:** COFs RIO‐54 and RIO‐55 have been reported in the literature and were obtained through solvothermal methods [21]. A glass pressure vessel was charged with the respective aldehyde (1 equivalent, 2 mmol). The top of the tube was closed with a rubber septum, and it atmosphere was changed by argon flow. After that, Bismarck Brown Y (0.75 equivalent, 1.5 mmol, 0.582 g), dioxane (13.6 mL), and a 6 M solution of acetic acid (4.6 mL) were added. The tube was closed with a PTFE cap and heated at 120 °C for 72 h. The resulting solid was vacuum filtered at room temperature, washed with ethanol and THF. Then, the obtained product was washed in ethanol in a Soxhlet extractor for three days. RIO‐54 (0.6371 g) and RIO‐55 (1.0626 g) were obtained as a brown solid.

### 2.2 Material Characterization Methods

**Powder X‐ray Diffraction (PXRD):** PXRD patterns were recorded in a Shimadzu XRD7000 diffractometer, using Cu‐K radiation with a 2 = 0.02° in the 2° to 90° 2 range.

**Fourier Transform Infrared (FTIR):** The infrared spectra in the 400–4000 cm^−1^ region was recorded in samples prepared as diluted KBr pellets using a FTLA‐2000 spectrometer.

**Textural Properties:** To analyze the textural properties (specific surface area, porosity) of the nanomaterials, low‐temperature nitrogen adsorption–desorption isotherms were recorded using a Quantachrome Nova 1200 adsorption analyzer or Autosorb 6200 MP. The surface area (SBET) was calculated according to the standard Brunauer–Emmett–Teller (BET) method. The total pore volume Vp was calculated from the nitrogen adsorption data at relative pressure P/P0 0.98–0.99. To calculate the pore size distributions (PSDs) in respect to SBET and pore volume (Vp), the data from the desorption branch were used. The proposed computational method is based on a regularization procedure under non‐negative condition for the PSD function (f(Rp) > 0 at any pore radius Rp), at fixed regularization parameter = 0.01. The pores as the voids between spherical particles randomly packed in aggregates (i), slit‐shaped (ii) and cylindrical pores (iii) were examined. This approach based on the Nguyen‐Do method [22, 23] was modified to be applied for various nanomaterials [24, 25, 26, 27]. The volume PSD function (fv(Rp)) and surface area PSD function (fs(Rp)) were used to estimate contributions of different types of pores (classified according to their sizes and shapes) to total pore volume (Vp) and SBET: nanopores (Vmicro, Smicro at Rp < 1 nm), mesopores (Vmeso, Smeso at 1 Rp 25 nm), and macropores (Vmacro, Smacro, at Rp > 25 nm) [28, 29].

**Scanning Electron Microscope (SEM):** The samples for SEM were inserted into the sample holder with carbon tape and then a conductive layer of gold was deposited. A SEM of the Tescan brand, model Vega 3, was used. The elemental chemical composition was determined through energy‐dispersive spectroscopy (EDS) probe, using a Bruker Nano XFlash Detector.

**Theoretical Powder X‐ray Diffraction (PXRD):** The theoretical PXRD was obtained using VESTA (Visualization for Electronic and Structural Analysis) software, using the CIF (Crystallographic Information File) of the COFs generated through theoretical calculations of the optimized structure. Optimization calculations of the model structures were performed using density functional theory (DFT) calculations under periodic boundary conditions with the Quantum Espresso code version 7.4.1 [30, 31]. The exchange and correlation effects were treated with the generalized gradient approximation (GGA) of the Perdew–Burke–Ernzerhof (PBE) functional, and the nuclei and electrons of the core were described by ultrasmooth pseudopotentials [32]. Van der Waals interactions were treated with the D3 correction method of Grimme et al. [33]. The Kohn–Sham orbitals were expanded on a plane wave basis set with a kinetic energy cutoff of 50 Ry and 400 Ry for the charge density. The first Brillouin zone integrations were performed with gamma‐centered Monkhorst–Pack k‐point sampling [34]. The atomic positions and cell parameters were optimized simultaneously until the forces acting on the atoms were less than 10–8 Ry Bohr‐1.

### 2.3 Enzyme Immobilization in COFs

The lipase immobilization, specifically *Diutina rugosa* lipase (Candida rugosa lipase) (DrL) obtained from Sigma–Aldrich (Catalog Number L‐1754), was achieved through a straightforward adsorption process. The following steps outline the procedure employed for successfully immobilizing lipase onto COFs. Initially, 10 mg of DrL was carefully dissolved in 4.5 mL of a 50 mM phosphate buffer solution (pH 6.8–7.0). Then, 10.0–10.9 mg of COFs were weighed into a 2.0 mL‐capacity tube, in triplicate or more replicates. Subsequently, for the standard test, 1.5 mL of the same 50 mM phosphate buffer solution (pH 6.8–7.0) and 200 μL of the prepared DrL solution was added to the tube. Other enzyme and buffer volumes were used to prepare the adsorption isotherms but always respecting the maximum volume of 1.7 mL, as described in the text. The mixture was subjected to an incubation period lasting 19 h at a controlled temperature of 17 °C at the standard test, and at room temperature (26 °C) as explained at the main text. Then, the content was centrifuged and the supernatant reserved for protein quantification of the unbound lipase. The pellet was washed five times with 500 μL of the same buffer to remove nonattached lipases and to confirm no leaching. The pellet was then resuspended in 40 mL of buffer as a use solution for activity measurements. In summary, the washing procedure consisted of five consecutive cycles, totaling 2.5 mL of buffer, performed as follows: 500 microliters of buffer were added to the Eppendorf tube containing the DrL@COF composite. The tube was sealed and vortexed for 10 s, followed by centrifugation at 2500 × g and 25 °C for 5 min. The supernatant was carefully separated and subsequently analyzed for total protein content, whereas the remaining pellet received a fresh 500 microliters aliquot of buffer to initiate the next washing cycle. The choice of buffer pH and temperature followed established protocols for DrL to ensure a balance between immobilization efficiency and protein stability. The pH was selected to match the enzyme's optimal stability range, preserving its native conformation and controlling the ionization of functional groups necessary for binding to the support. Regarding the temperature settings (17 and 26 °C), these were chosen to provide a controlled environment that favors immobilization kinetics while avoiding thermal denaturation.

### 2.4 Protein Quantification

Protein quantification of the unbound lipase, following the enzyme immobilization process, was carried out using the Bradford method (Bradford, 1976). We used the Quick Start Bradford Protein Assay Kit (Catalog Number 5000201) from Biorad, and the 2.2 Microassay Protocol (in a 96‐well plate), with a linear range of 1.25–10 µL except for, that a 50 µL of each sample, was mixed to 100 µL of the Bradford reagent provided by the kit. Following incubation for 5 min at room temperature, the absorbance of each sample was measured at 595 nm using a Molecular Devices Spectramax M2e spectrophotometer. A standard curve was created by plotting the 595 nm absorbance values (on the *y*‐axis) against the known concentrations in µg of bovine serum albumin (BSA) solutions.

### 2.5 Measurement of Lipase Activity

The production of 4‐methylumbelliferone, from 4‐ methylumbelliferyl heptanoate (MUF‐HEPT), was accessed by fluorescence, under the initial reaction conditions [17]. Standard curves were constructed using solutions ranging from 82.8 to 1225.00 μM of MUF dissolved in a 50 mM sodium phosphate buffer at pH 6.8–7.0. All experiments were conducted in triplicate (*n* = 3) or more. The catalysis was made in a 125 mL Erlenmeyer, containing 100 µL of use‐solution of DrL@COF and 9.9 mL MUF‐HEPT at 100 μM, also diluted at the same buffer. Aliquots were removed every 2 min, centrifuged, and the supernatant was measured by its fluorescence (λex = 323 nm; λem = 448 nm), in Molecular Devices Spectramax M2e. One unit of total activity was defined as the quantity of enzyme required to produce 1 nmol of MUF per minute under the test conditions and was expressed in micromoles of MUF/min (µmoles/min). Specific activity was defined as the quantity of enzyme required to produce 1 nmol of MUF per minute per microgram of enzyme under the test conditions and was expressed in micromoles of MUF/min.µg of enzyme (µmol min^−1^ mg^−1^). The natural substrate degradation of MUFHEPT was monitored and subtracted for activity calculations.

## 3 Results and Discussion

To establish a robust platform for the immobilization of *Diutina rugosa* lipase (DrL), four distinct COFs were strategically synthesized utilizing readily available building blocks. The materials were initially selected for their hydrophobic character, which provides good affinity for the enzyme and creates a microenvironment favorable to lipase activity [35].

The synthesized materials were RIO‐12, RIO‐13, RIO‐54, and RIO‐55. RIO‐12 and RIO‐13 are microporous COFs constructed from hydrazine and the aldehydes triformylresorcinol and triformylphloroglucinol, respectively. In contrast, RIO‐54 and RIO‐55 are mesoporous COFs synthesized by reacting the dye Bismarck Brown Y with triformylresorcinol and triformylphloroglucinol, respectively (Figure 1). Within each pair, the COFs exhibit similar functional groups but markedly different specific surface areas, as observed for both RIO‐12/RIO‐13 and RIO‐54/RIO‐55 (Table 1). These similarities and differences provide a basis for investigating how framework chemistry and textural properties influence enzyme immobilization and catalytic activity.

**FIGURE 1:**
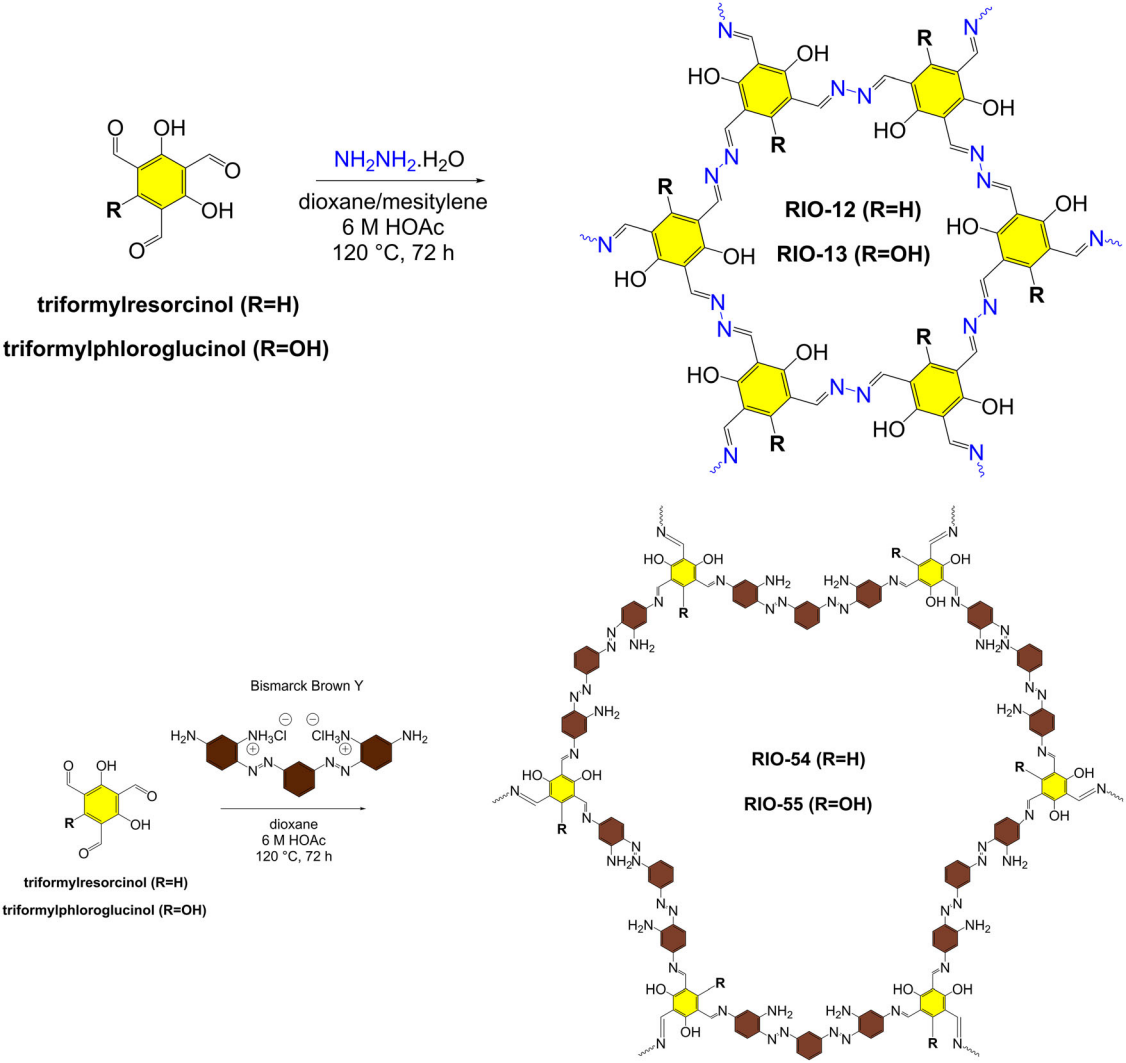
Schematic representation of the synthetic routes employed to prepare the four covalent organic frameworks (RIO‐12, RIO‐13, RIO‐54, and RIO‐55) used in this study. The COFs were obtained by reacting hydrazine or Bismarck Brown Y with the corresponding aldehyde precursors under solvothermal conditions.

**TABLE 1:** Textural properties of the synthesized COFs, including BET‐specific surface area and average pore diameter, together with enzyme loading and specific activity values for the corresponding DrL–COF composites. These data allow comparison of the textural characteristics of the supports alongside their enzyme immobilization efficiencies and catalytic activities.

COF	**Enzyme loading** a, %	Enzyme loading, µg	**Specific surface area (S**_**BET**_ **),** m^2^g^−1^	Pore size, Å	Specific activity, U mg^−1^
RIO‐12	99.8	3.71	1164	11	408.5 ± 7.9
RIO‐13	99.8	3.26	384	11	235.4 ± 62.6
RIO‐54	99.9	2.56	35	34	93.3 ± 5.7
RIO‐55	99.9	2.22	280	34	24.4 ± 0.7

a Protein quantification was performed using the Bradford method, as described in Section 2.4.

The materials were synthesized via solvothermal methods, as previously reported [19, 21]. Characterization of the COFs was performed using Fourier‐transform infrared spectroscopy (FTIR), N_2_ sorption, SEM, and PXRD.

FTIR analysis revealed bands assigned to C═N stretching vibrations at approximately 1693 and 1596 cm^−1^ for RIO‐12 and RIO‐13, respectively (Figure 2). The positions of these bands have been attributed to the conjugated environment of the C═N linkages within the frameworks [19]. Additionally, the band broadening and shift toward lower wavenumbers observed for RIO‐13, the more hydroxylated material, suggest stronger intramolecular hydrogen bonding [19]. For RIO‐54 and RIO‐55, bands at 1627 and 1619 cm^−1^, respectively, were assigned to C = O stretching vibrations associated with keto‐enamine units, while bands in the 2800–3100 cm^−1^ region were assigned to N─H stretching vibrations (Figure 2). Bands at 1448 and 1438 cm^−1^, respectively, were attributed to vibrations involving the azo (─N═N─) moiety of Bismarck Brown Y, supporting the retention of this group within the frameworks (Figure 2) [21].

**FIGURE 2:**
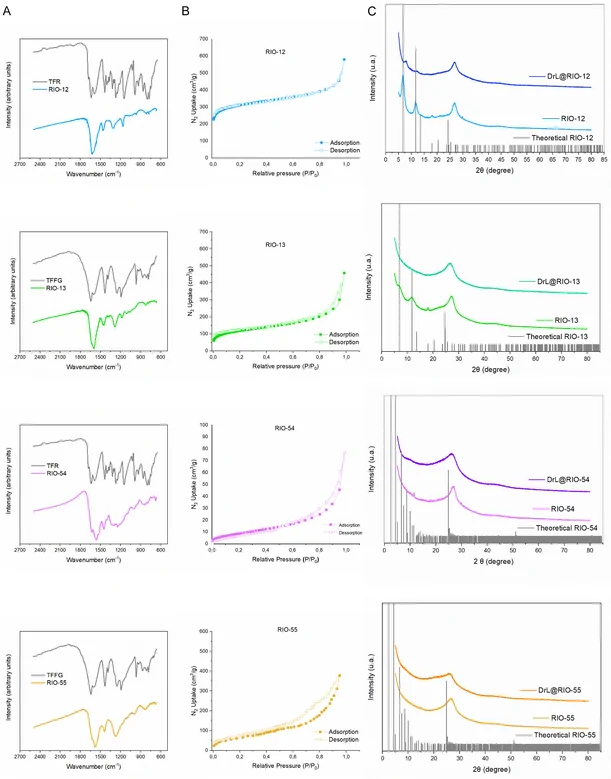
Characterization of the four synthesized COFs: (A) Fourier‐transform infrared (FTIR) spectra showing the characteristic vibrational bands associated with the framework structures; (B) N_2_ adsorption–desorption isotherms illustrating differences in the adsorption behavior and textural properties of the materials; and (C) powder X‐ray diffraction (PXRD) patterns of the COFs before and after lipase immobilization, allowing assessment of framework structural integrity following enzyme adsorption.

The N_2_ adsorption–desorption isotherms of RIO‐12 and RIO‐13 exhibited type I profiles, characteristic of microporous materials (Figure 2). The specific surface areas calculated using the BET method were 1164 and 384 m^2^ g^−1^ for RIO‐12 and RIO‐13, respectively. Further analysis using the de Boer t‐plot method yielded micropore surface areas of 781 and 177 m^2^ g^−1^, external surface areas of 262 and 236 m^2^ g^−1^, and micropore volumes of 0.363 and 0.089 cm^3^ g^−1^, respectively. These results indicate that RIO‐12 has a substantially greater contribution from microporosity than RIO‐13, whereas their external surface areas are comparatively similar.

RIO‐54 exhibited an N_2_ adsorption isotherm described as type III (Figure 2), whereas the adsorption–desorption isotherm of RIO‐55 displayed hysteresis, suggesting the presence of mesoporosity. The larger pore dimensions reported for these materials, compared with RIO‐12 and RIO‐13, may be associated with the more extended structure of the Bismarck Brown Y building block. Despite being prepared using the same dye, RIO‐54 and RIO‐55 exhibited markedly different reported BET specific surface areas of 35 and 280 m^2^ g^−1^, respectively, as previously reported [21].

Figure 2 shows the calculated and experimental powder X‐ray diffraction (PXRD) patterns of RIO‐12, RIO‐13, RIO‐54, and RIO‐55. For RIO‐12 and RIO‐13, the main diffraction peaks were observed at approximately 2θ = 6°, 11°, and 27°, assigned to the (100), (210), and (002) reflections, respectively, with the latter associated with layer stacking [19]. RIO‐54 and RIO‐55, in contrast, exhibited a broad diffraction peak centered at 2θ ≈ 28°, corresponding to an interplanar spacing of approximately 3.2 Å and consistent with stacking between the two‐dimensional layers [21]. The low crystallinity of these frameworks has been attributed to the nonlinearity and low symmetry of their building blocks, which may hinder regular framework assembly and layer stacking, favoring stacking faults and the formation of disordered regions [21]. Such structural disorder may also contribute to the development of meso‐ and macroporous regions.

Following the characterization of the COF supports, DrL immobilization was investigated. DrL was selected because of its high catalytic efficiency in both hydrolysis and esterification reactions [21]. Furthermore, it has been widely used as a model enzyme in lipase immobilization studies owing to its extensive characterization in both free and immobilized forms [36, 37]. However, DrL is known to undergo aggregation in aqueous media, which can significantly affect its stability, catalytic activity, and substrate specificity, thereby limiting the practical use of crude enzyme preparations [38]. Immobilization may help mitigate these limitations by restricting enzyme aggregation and providing a more favorable local microenvironment, potentially improving the physicochemical and catalytic properties of DrL and expanding its practical applicability [39].

Enzyme immobilization can be achieved through a variety of physical and chemical methods. However, for industrial and commercial applications, practical and operationally simple approaches are particularly desirable. An ideal immobilization platform should therefore operate under mild conditions, require only a few processing steps, and minimize the use of additional reagents [16, 17]. In this context, physical adsorption is especially attractive because it can provide a simple route to enzyme immobilization without requiring chemical modification of either the enzyme or the support.

Initially, the immobilization process was carried out by treating approximately 10 mg of the material with the enzyme in a 50 mM phosphate buffer solution (pH 7) at 17 °C for 19 h. Protein quantification of the unbound lipase after the immobilization process was determined using the Bradford method [40]. Analysis of the supernatant before and after immobilization revealed a consistently high loading efficiency, ranging from 99.8% to 99.9% (Table 1), which is notably higher than values reported in previous studies on lipase immobilization [3, 39]. For each support material, a protein binding of approximately 43.935 ± 0.023 mg/g was achieved after 19 h at 17 °C.

After enzyme immobilization, the PXRD patterns showed a slight decrease in the intensity and definition of the characteristic COF reflections (Figure 2), while FTIR analysis revealed no substantial changes in the characteristic functional groups of the frameworks. The incorporation of guest species, such as ions or molecules, into layered materials can affect their structural organization and may introduce local distortions or defects [41, 42, 43]. In the present systems, interactions between the enzyme and the COF surface or interlayer regions may partially disturb the ordered arrangement of the two‐dimensional layers, thereby contributing to the observed changes in the diffraction patterns. Nevertheless, attenuation of the reflections may also result from enzyme deposition on the COF particles or changes in diffraction contrast. Therefore, the PXRD results indicate a modest alteration in framework ordering rather than extensive structural degradation, whereas the preservation of the characteristic FTIR bands suggests that the chemical structure of the COFs remained largely unchanged after immobilization.

Finally, SEM images acquired before and after immobilization showed no substantial changes in the overall particle morphology or surface appearance of the COFs (Figure S1). This result indicates that enzyme adsorption did not produce morphological alterations detectable at the length scale and resolution of the SEM analysis, in agreement with the preservation of the framework characteristics observed by FTIR and PXRD.

Given the high immobilization efficiencies obtained under the initial conditions and the limited number of systematic studies addressing enzyme adsorption kinetics on COFs, we subsequently investigated the adsorption behavior of the four supports by monitoring the decrease in protein concentration in the aqueous phase over time. Experiments performed at 26 °C reproduced the high protein uptake previously observed after incubation at 17 °C for 19 h. Remarkably, the protein concentration in solution decreased to its final measured level within the first 10 s for all four COFs, demonstrating that DrL uptake was extremely rapid under the experimental conditions employed (Figure 3). No clear differences in adsorption behavior among the supports could be resolved within this time interval, despite their markedly different BET specific surface areas and pore diameters. These findings suggest that neither the total surface area accessible to N_2_ nor the nominal pore size was the primary factor controlling the initial uptake of DrL. Instead, the shared physicochemical characteristics of the COF surfaces, particularly their organic and aromatic character, may provide similarly favorable environments for rapid enzyme adsorption. Comparable effects of surface chemistry on the initial adsorption behavior of albumin and fibrinogen on different surfaces were reported by Wertz and Santore [44].

**FIGURE 3:**
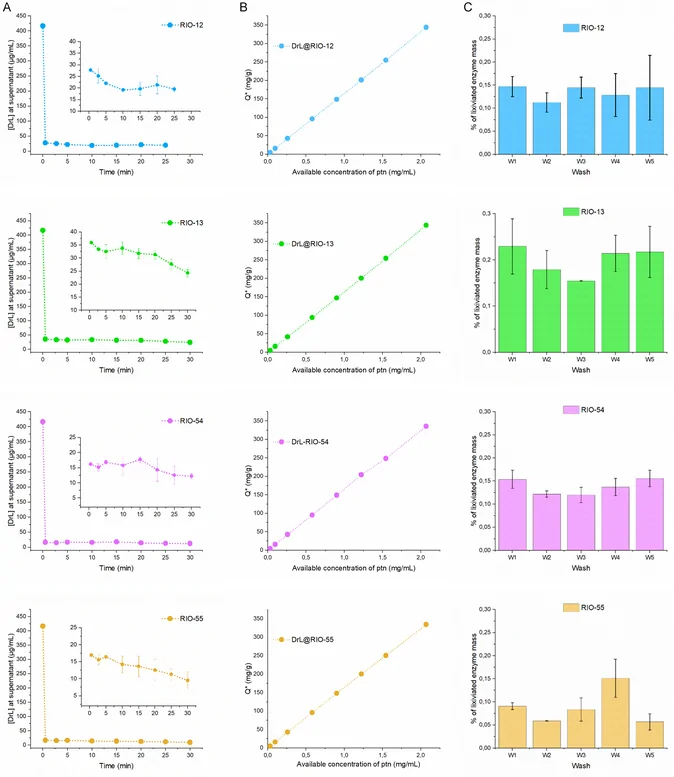
(A) Kinetics of DrL adsorption onto the different COFs. The inset shows the same dataset after excluding the first point, allowing differences among the subsequent measurements to be visualized more clearly. (B) DrL adsorption isotherms obtained for the different COFs. All error bars correspond to standard deviations and are smaller than 0.67 mg g^−1^. (C) Percentage of immobilized enzyme released during successive washing cycles, showing minimal enzyme leaching from the DrL–COF composites. Error bars represent standard deviations.

Furthermore, the rapid uptake observed for all four COFs is consistent with a high affinity between DrL and the predominantly organic surfaces of the supports (Figure 3). Hydrophobic interactions may contribute substantially to this behavior because adsorption of proteins onto hydrophobic interfaces is generally favored by the displacement of interfacial water and by interactions between nonpolar regions of the protein and the support surface [45]. Following the initial contact, conformational rearrangements and spreading of the adsorbed protein may further increase the number of enzyme–surface contacts, strengthening its association with the support. Previous studies have shown that these processes can occur more readily on hydrophobic than on hydrophilic surfaces, resulting in rapid and persistent protein adsorption [45]. Although the present kinetic results are compatible with this mechanism, additional experiments using supports with systematically varied surface hydrophobicity would be required to establish the relative contribution of hydrophobic interactions.

In addition to indicating a high affinity between the COFs and DrL, the rapid immobilization observed in this study represents an important operational advantage. Reducing the immobilization time from several hours to seconds could increase process throughput, decrease equipment occupancy, and simplify biocatalyst preparation. Shorter processing times may also reduce energy consumption and limit prolonged exposure of the enzyme to conditions that could promote loss of activity. Therefore, the nearly instantaneous uptake of DrL, combined with the operational simplicity of physical adsorption, reinforces the potential of these COFs as practical supports for enzyme immobilization.

To further investigate the adsorption process and determine how protein uptake varied with enzyme concentration, adsorption isotherms were obtained for DrL on the four COFs (Figure 3B). Adsorption isotherms provide valuable information about the relationship between the equilibrium concentration of an adsorbate in solution and the amount retained by a solid support. In enzyme immobilization studies, this approach can therefore help evaluate the affinity and loading behavior of the support and identify possible differences in enzyme–surface interactions. Such information is relevant both to understanding the immobilization process and to defining suitable conditions for the preparation of enzyme‐based biocatalysts.

The initial protein concentration in the medium ranged from 0.032 to 2.07 mg mL^−1^, with eight concentrations investigated: 0.032, 0.098, 0.259, 0.580, 0.901, 1.223, 1.544, and 2.071 mg mL^−1^. Under all conditions investigated, more than 99% of the enzyme was removed from the aqueous phase by each of the four COFs, including at the highest initial DrL concentration tested, which was limited by the maximum enzyme concentration experimentally attainable in the buffer solution. Accordingly, the amount of enzyme retained by the supports increased approximately linearly throughout the investigated concentration range, and no adsorption plateau was observed (Figure 3B). Within this experimental range, the profiles could be described by a Henry‐type relationship, in which the amount adsorbed increases proportionally with the equilibrium enzyme concentration. This behavior indicates that the adsorption capacity of the COFs was not saturated under the conditions employed and that additional adsorption sites remained available as the initial enzyme concentration increased.

A Henry‐type regime corresponds to the low‐concentration region of an adsorption isotherm, where the adsorbed amount is approximately proportional to the equilibrium concentration and saturation effects are negligible. It is analogous to the low‐pressure limit of gas adsorption, in which the Langmuir equation approaches a linear relationship between adsorbate uptake and equilibrium pressure [46]. In this study, however, the absence of a plateau indicates only that saturation was not reached within the experimentally accessible DrL concentration range; it does not establish the maximum adsorption capacity or, by itself, demonstrate monolayer formation. The high uptake observed for all four COFs is nevertheless consistent with favorable enzyme–support interactions, which may include hydrophobic, electrostatic, and dispersion forces.

The stability of the DrL@COF composites was subsequently evaluated by monitoring enzyme release during successive washing cycles (Figure 3C). For all four composites, the amount of protein detected in each washing fraction remained below 0.3% of the initially immobilized enzyme mass. These low values indicate that DrL was strongly retained by the COF under the washing conditions employed, despite immobilization being based on physical adsorption. Among the materials, DrL@RIO‐13 showed the highest enzyme release, followed by DrL@RIO‐55, although leaching remained low in both cases.

Finally, the catalytic performance of the resulting biocatalysts was evaluated by comparing the activity of the COF‐immobilized enzymes with that of free DrL in the hydrolysis of 4‐methylumbelliferyl heptanoate (MUF‐HEPT), using a fluorimetric assay (Figure 4 and Table 1). The specific activities determined for free DrL, DrL@RIO‐12, DrL@RIO‐13, DrL@RIO‐54, and DrL@RIO‐55 were 27.7, 408.5, 235.4, 93.3, and 24.4 U mg^−1^ of enzyme, respectively. One unit of enzymatic activity (U) was defined as the amount of enzyme that catalyzes the formation of 1 µmol of product per minute under the assay conditions. Specific activities were normalized to the protein mass in the free‐enzyme preparation or immobilized on the support, rather than to the total mass of the enzyme–COF composite. The catalytically active protein fraction was not independently determined in either preparation; therefore, the reported values represent apparent specific activities per unit protein mass and do not establish catalytic turnover per active enzyme molecule.

**FIGURE 4:**
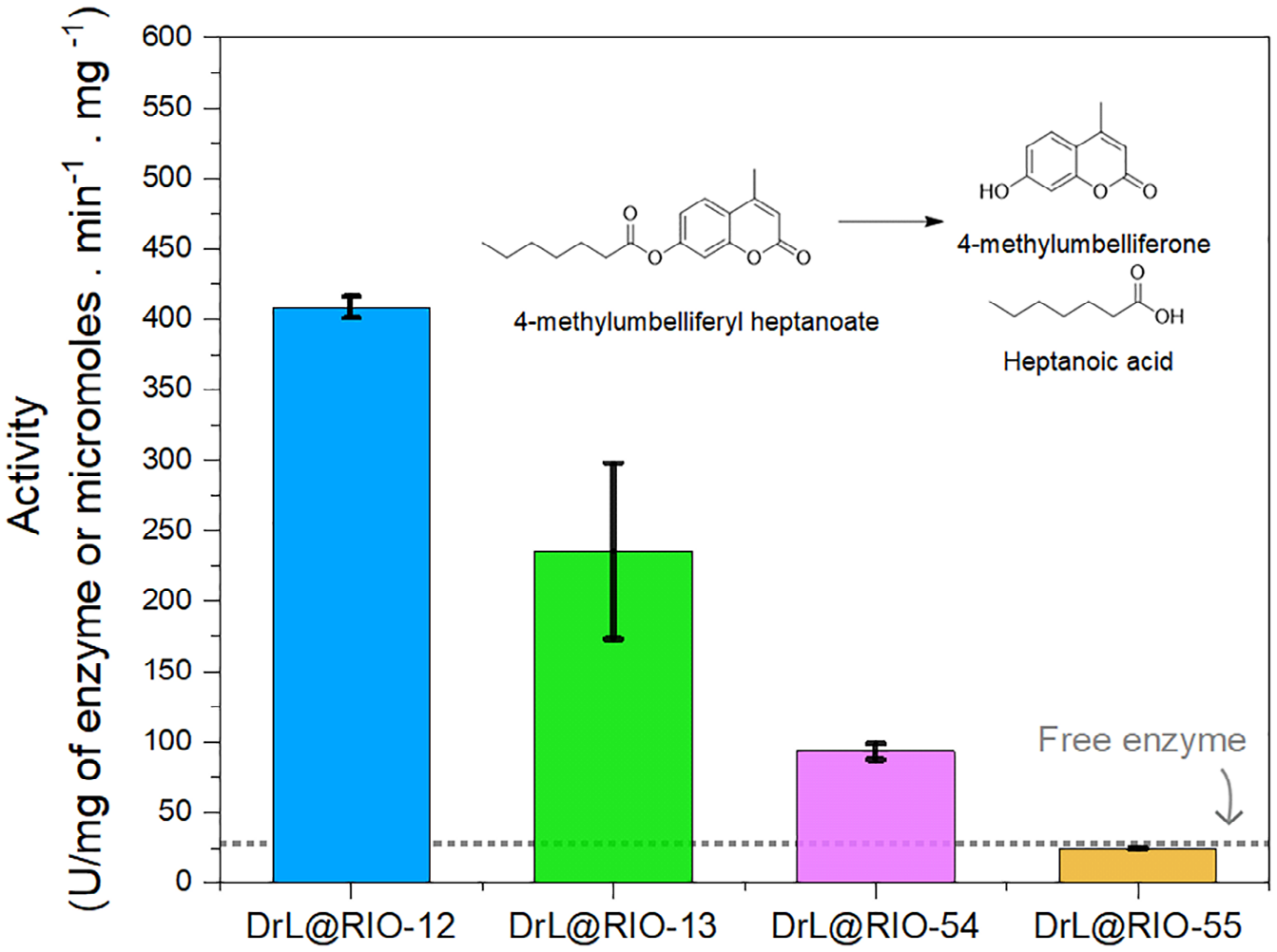
Specific activities of *Diutina* (*Candida*) *rugosa* lipase (DrL) immobilized on four different COFs: RIO‐12, RIO‐13, RIO‐54, and RIO‐55. The dashed line indicates the specific activity of free DrL under the same assay conditions. Error bars represent standard deviations.

Interpretation of these results should take into account that immobilization on hydrophobic supports can affect lipase activity in different ways. Strong enzyme–surface interactions may promote conformational deformation, unfavorable orientation, or steric restrictions that reduce substrate accessibility to the active site [47]. Conversely, hydrophobic interfaces can stabilize the open, catalytically accessible conformation of lipases through interfacial activation, potentially increasing their apparent activity [48].

In a seminal study, Palomo et al. immobilized lipases from *Candida antarctica* B, *Mucor miehei*, and *Candida rugosa* on octadecyl–Sepabeads through interfacial adsorption. The authors attributed the observed hyperactivation to stabilization of the open conformation on the hydrophobic support and reported a specific activity up to 20 times higher than that of the soluble enzyme for the *M. miehei* derivative [49]. The influence of hydrophobic support characteristics on lipase catalytic properties was also investigated by Fernández‐Lorente et al. [50]. Furthermore, Sun, Zhu, and Matyjaszewski reported an activity enhancement of up to 40‐fold relative to the free lipase under their optimal conditions, using core–shell nanoparticles designed to promote and protect an active lipase conformation [51].

Enhanced enzymatic performance has also recently been reported using COFs. Xing et al. incorporated flexible oligo(ethylene oxide) chains into COF channels to create artificial hydration‐like microenvironments that supported enzyme function in organic media. At 0% relative humidity, the encapsulated enzyme achieved a conversion approximately 13 times that obtained with the free enzyme [52] . Although these systems operate through different microenvironmental effects, they illustrate how support chemistry and the organization of the enzyme surroundings can jointly influence catalytic performance.

For the COFs investigated here, differences in the balance between aromatic and polar structural features provide a basis for considering the possible contribution of hydrophobicity. Tan et al. described hydrophobic pore environments in aromatic COFs in terms of an aromatic C─H “carpet,” while also highlighting the influence of polar sites and their accessibility on interactions with water [53] . Furthermore, beyond chemical composition, the local surface environment can also be influenced by the arrangement of adjacent framework layers. Stacking disorder and interlayer offsets have been experimentally observed in layered COFs and can affect pore geometry and the accessibility of functional sites [54]. Such structural variations could therefore modulate DrL–COF interactions by altering the spatial distribution and accessibility of the surface groups encountered by the enzyme.

In the present materials, the greater aromatic content associated with the Bismarck Brown Y‐derived units in RIO‐54 and RIO‐55 could favor a more hydrophobic environment than that provided by RIO‐12 and RIO‐13. Likewise, the lower oxygen content of frameworks prepared from triformylresorcinol, relative to their triformylphloroglucinol‐derived counterparts, could decrease their affinity for water. These structural considerations suggest a tentative increase in hydrophobic character in the order RIO‐13 < RIO‐12 < RIO‐55 < RIO‐54. However, this sequence should be regarded as a working hypothesis, since the effective hydrophobicity of the enzyme‐contacting surface also depends on the chemical state, spatial distribution, and accessibility of polar groups.

Notably, this proposed hydrophobicity sequence does not reproduce the measured activity ranking, DrL@RIO‐12 > DrL@RIO‐13 > DrL@RIO‐54 > DrL@RIO‐55. Relative to free DrL, immobilization increased the specific activity by approximately 14.8‐, 8.5‐, and 3.4‐fold on RIO‐12, RIO‐13, and RIO‐54, respectively, whereas DrL@RIO‐55 retained approximately 87% of the free‐enzyme activity. Although the higher activity of DrL@RIO‐12 relative to DrL@RIO‐13, and of DrL@RIO‐54 relative to DrL@RIO‐55, is consistent with the proposed hydrophobicity differences within each pair, this relationship does not extend across the entire series. In particular, the Bismarck Brown Y‐derived frameworks, despite their potentially greater hydrophobic character, yielded lower specific activities than RIO‐12 and RIO‐13. Thus, hydrophobicity alone does not account for either the magnitude or the direction of the activity changes induced by immobilization.

The textural properties of the COFs provide a complementary perspective on the observed differences in enzymatic activity. As discussed above, RIO‐12 and RIO‐13 contain predominantly microporous domains, with reported pore diameters of approximately 11 Å, whereas RIO‐54 and RIO‐55 exhibit larger pores, with reported diameters of approximately 34 Å. The hydrodynamic dimensions reported for DrL are in the range of 50–80 Å [55], exceeding the nominal pore diameters of all four COFs. The enzyme is therefore unlikely to enter the intrinsic framework pores without substantial structural deformation or local framework flexibility. Consequently, DrL adsorption is expected to occur predominantly on the external surfaces of the COF particles and within larger interparticle voids or structural defects accessible to the enzyme (Figure 5).

**FIGURE 5:**
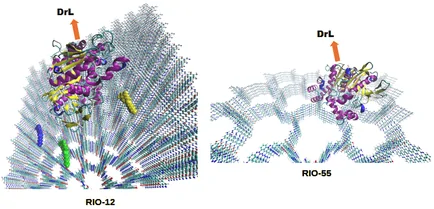
Comparison between the pore size of COFs and the molecular dimensions of DrL for composites DrL@RIO‐12 (left) and DrL@RIO‐55 (right).

RIO‐12 and RIO‐13, which produced the composites with the highest catalytic activities, have closely related framework structures and similar nominal pore sizes but differ substantially in their BET specific surface areas. RIO‐12 exhibits a specific surface area of 1164 m^2^ g^−1^, approximately three times that of RIO‐13 (384 m^2^ g^−1^). This difference parallels the higher specific activity of DrL@RIO‐12 relative to DrL@RIO‐13 and suggests that the greater surface area of RIO‐12 may contribute to the catalytic performance of the composite. Nevertheless, this relationship should not be considered independently of differences in framework chemistry, surface functionality, and enzyme–support interactions, which may also influence the conformation and microenvironment of the immobilized enzyme. The comparison between RIO‐54 and RIO‐55 further highlights the contribution of factors beyond BET surface area to catalytic performance. Despite having the lowest surface area (35 m^2^ g^−1^), RIO‐54 yielded a composite with a specific activity approximately 3.9 times that of DrL@RIO‐55, whose support exhibits a surface area of 280 m^2^ g^−1^. This contrast is particularly informative because both materials have the same reported pore size (34 Å). The comparatively high activity of DrL@RIO‐54 is therefore consistent with a favorable contribution from the local enzyme–support environment, although this comparison alone does not establish hydrophobicity as the underlying cause.

Sasaki et al. (2025) compared the catalytic performance of Amano Lipase PS immobilized on a microporous COF, TUS‐39, with a pore diameter of 0.9 nm, and a mesoporous COF, TUS‐64, with a pore diameter of 4.7 nm [56]. Because the enzyme could not enter the smaller pores of TUS‐39, it was predominantly immobilized on the external surface, where the active site remained more accessible to the substrate, resulting in higher catalytic activity. In contrast, immobilization within the larger pores of TUS‐64 provided greater protection against denaturation but imposed diffusional and accessibility limitations, leading to slightly lower conversion than that obtained with the surface‐immobilized enzyme. These findings illustrate that larger pores and internal enzyme encapsulation do not necessarily result in higher catalytic activity, as the final performance reflects a balance between enzyme protection and substrate accessibility.

To rationalize the differences in catalytic performance, it is important to consider that the apparent activity of an immobilized enzyme depends not only on its intrinsic catalytic properties but also on mass transfer and substrate accessibility. The overall process involves transport of the substrate from the bulk solution to the enzyme microenvironment, binding at the active site, chemical conversion, and product release and diffusion back into the bulk medium. The support can therefore influence activity through interactions not only with the enzyme but also with the substrate and product. When the support preferentially concentrates the substrate in the vicinity of the immobilized enzyme, the local substrate concentration may become higher than that in the bulk solution. This phenomenon, commonly described as a positive partition effect, can enhance the apparent catalytic activity without necessarily altering the intrinsic catalytic rate of the enzyme. In the present system, hydrophobic interactions between the COF surface and the nonpolar region of MUF‐HEPT may favor substrate accumulation near the immobilized DrL, thereby increasing the frequency of productive enzyme–substrate encounters.

To further examine whether a substantial surface area remained available after enzyme immobilization, the textural properties of the DrL@RIO‐12 composite were evaluated by N_2_ adsorption–desorption analysis (Figure 6). The composite exhibited a BET specific surface area of 828.2 m^2^ g^−1^, compared with 1164 m^2^ g^−1^ for pristine RIO‐12. This decrease may result from deposition of the enzyme on the external surface, partial obstruction of pore entrances, or the additional mass contributed by the comparatively nonporous protein phase. Nevertheless, DrL@RIO‐12 retained approximately 71% of the original BET specific surface area, demonstrating that a substantial fraction of the framework porosity remained accessible to N_2_ after immobilization. The preservation of this high apparent surface area is compatible with the proposed partition effect, since the COF may retain sufficient accessible surface to interact with the substrate in the vicinity of the immobilized enzyme. However, because N_2_ adsorption is measured in the dry state and with a probe molecule much smaller than MUF‐HEPT, these results provide indirect rather than definitive evidence of substrate enrichment under the aqueous reaction conditions.

**FIGURE 6:**
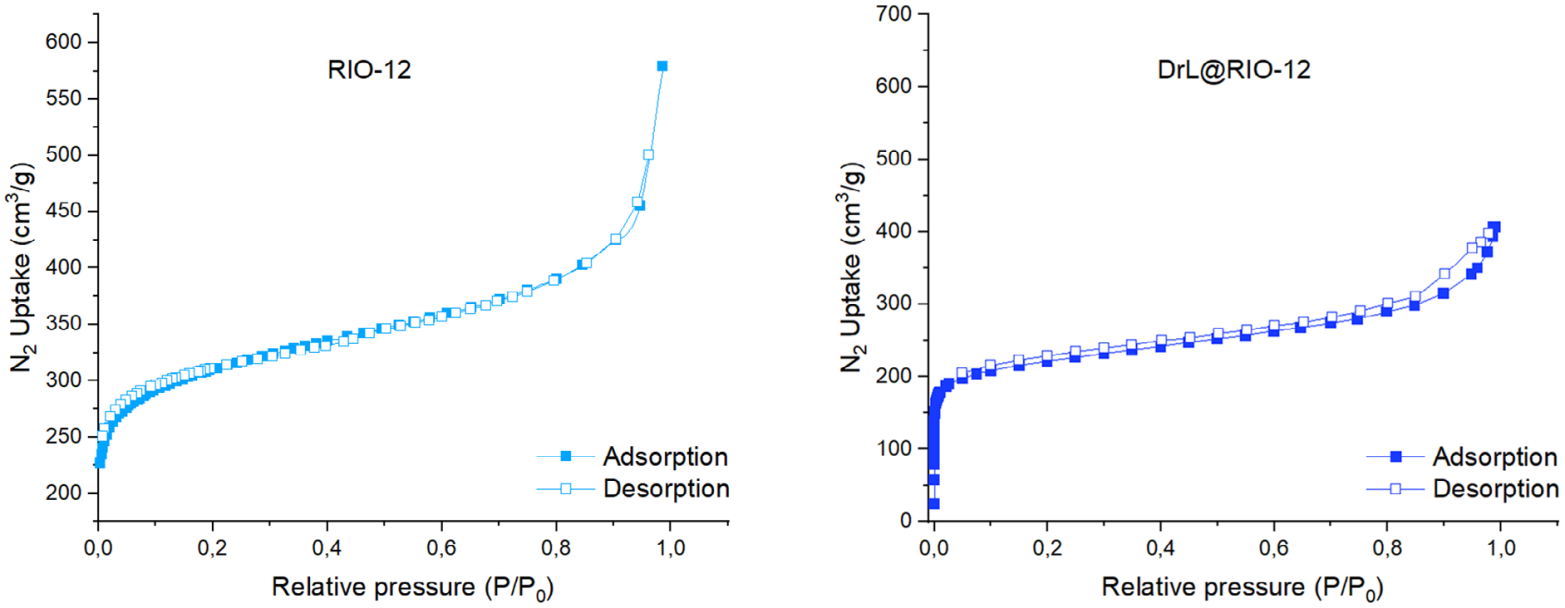
N_2_ adsorption–desorption isotherms of pristine RIO‐12 and the DrL@RIO‐12 composite, showing the changes in the textural properties of the material after enzyme immobilization.

Furthermore, substrate adsorption experiments showed that RIO‐12 retained MUF‐HEPT from the reaction medium (Figure S2), supporting the hypothesis that the COF can concentrate the substrate within its interfacial region. This preferential partitioning may increase the local substrate concentration near the immobilized enzyme and contribute to the enhanced apparent catalytic activity. Based on these findings, we propose that two complementary effects account for the activity enhancement observed for DrL@RIO‐12 and DrL@RIO‐13. First, the chemically heterogeneous surfaces of these COFs can establish multiple noncovalent interactions with the enzyme, including hydrophobic interactions with the aromatic framework and hydrogen bonding involving nitrogen‐ and oxygen‐containing groups. These interactions may promote strong enzyme retention while favoring an orientation or conformation in which the active site remains accessible to the substrate. Residual aldehyde groups, if present on the COF surface, could also interact with accessible amino groups of the enzyme, although the formation of covalent enzyme–support bonds was not directly demonstrated in this study. Second, the high surface area and organized nanoporous structure of RIO‐12 may favor a positive partition effect by concentrating MUF‐HEPT near the externally immobilized enzyme. The combination of favorable enzyme–surface interactions and local substrate enrichment therefore provides a plausible explanation for the particularly high apparent catalytic activity of DrL@RIO‐12.

## 4 Conclusion

This study demonstrated that RIO‐12, RIO‐13, RIO‐54, and RIO‐55 are effective supports for the immobilization of *Diutina rugosa* lipase. All four COFs provided more than 99% protein uptake within the first 10 s and enzyme leaching below 0.3% per washing cycle, indicating rapid adsorption and strong enzyme retention. The similar uptake observed despite substantial differences in surface area and pore size suggests that the organic and aromatic character of the COF surfaces plays an important role in DrL adsorption.

Immobilization on three of the four COFs enhanced DrL activity. DrL@RIO‐12 exhibited the highest specific activity, corresponding to a 14.8‐fold increase relative to the free enzyme, followed by DrL@RIO‐13 and DrL@RIO‐54. The results suggest that this enhancement arises from a combination of favorable enzyme–surface interactions, interfacial activation, preservation of active‐site accessibility, and substrate enrichment near the immobilized enzyme. In particular, the high surface area retained by DrL@RIO‐12 and its affinity for the substrate are consistent with a positive partition effect.

Overall, the rapid, single‐step immobilization, high enzyme retention, and substantial activity enhancement demonstrate the potential of hydrophobic COFs as practical supports for the development of highly active heterogeneous biocatalysts.

The scope of these conclusions should nevertheless be considered in light of the use of a single model substrate, MUF‐HEPT. Although this fluorogenic substrate provides a standardized basis for comparing the catalytic performance of free and immobilized DrL, the magnitude of the observed activity enhancements may depend on substrate properties and reaction conditions and should not be directly extrapolated to natural lipids or other transformations. Beyond these assay‐specific results, the comparative analysis supports a broader design principle: COF chemical composition alone is insufficient to predict catalytic performance, and even efficient enzyme uptake and strong retention do not necessarily translate into enhanced activity. These findings highlight the need to consider surface chemistry and textural properties together when developing COF‐based biocatalysts. Further studies underway in our laboratory are exploring lipid hydrolysis, biomass valorization, and continuous‐flow operation to assess the applicability of these findings under technologically relevant conditions.

## Funding

Fundação Carlos Chagas Filho de Amparo à Pesquisa do Estado do Rio de Janeiro.

## Conflicts of Interest

The authors declare no conflicts of interest.

## Supporting information

Supplementary Material

## Data Availability

The data that support the findings of this study are available in the Supporting Information of this article.
