# Supplementary material for: Hydrophobic Covalent Organic Frameworks as a Platform for Instantaneous and High‐Capacity Immobilization of Diutina (Candida) rugosa Lipase: A Simple Strategy to Boost Enzyme Activity
Source: Chempluschem. 2026 Nov 4;91(11):e70284. doi: 10.1002/cplu.70284 (PMC13736130; doi:10.1002/cplu.70284)
Supplement: Supplementary file 1 [file CPLU-91-e70284-s001.pdf]

Supporting Information

Hydrophobic Covalent Organic Frameworks as a Platform for Instantaneous and High-Capacity Immobilization of *Diutina (Candida) rugosa* Lipase: A Simple Strategy to Boost Enzyme Activity

Leticia Dobler[a]±, Mírian Lima de Almeida Lippi[a]±, Luiz Octávio Mateus Nogueira[a], Davi Forastieri Tostes[a], Alexandre da Silva França[a], Fábio Junior Ferreira da Silva Henrique[a], Leandro Sodré de Abreu[a], Gabriel Almeida Mendonça da Silva[a], Sidnei Moura e Silva[b], Cesar Aguzzoli[c], Rodrigo O. M. A. de Souza[a], Rodrigo Volcan Almeida[a], Pierre Mothé Esteves[a]*, Raoni Schroeder Borges Gonçalves[a]*

± Both authors contributed equally.

* corresponding authors

*pesteves@iq.ufrj.br, raoni.schroeder@iq.ufrj.br

[a]Instituto de Química, Universidade Federal do Rio de Janeiro, Brazil

[b]Departamento de Tecnologia, LBIOP – Laboratório de Biotecnologia de Produtos Naturais e Sintéticos, Instituto de Biotecnologia, Universidade de Caxias do Sul, Caxias do Sul, Brazil

[c]Programa de Pós-Graduação em Ciência e Engenharia de Materiais (PPGMAT), Universidade de Caxias do Sul (UCS), Francisco Getúlio Vargas 1130, Caxias do Sul, RS 95070-560, Brazil

1. Scanning Electron Microscopy

Figure S1. Scanning Electron Microscopy (SEM) images of the COFs before and after immobilization of *Diutina* (*Candida*) *rugosa* lipase (DrL). Morphological features such as surface roughness and particle size remain consistent, with slight changes indicating enzyme loading.

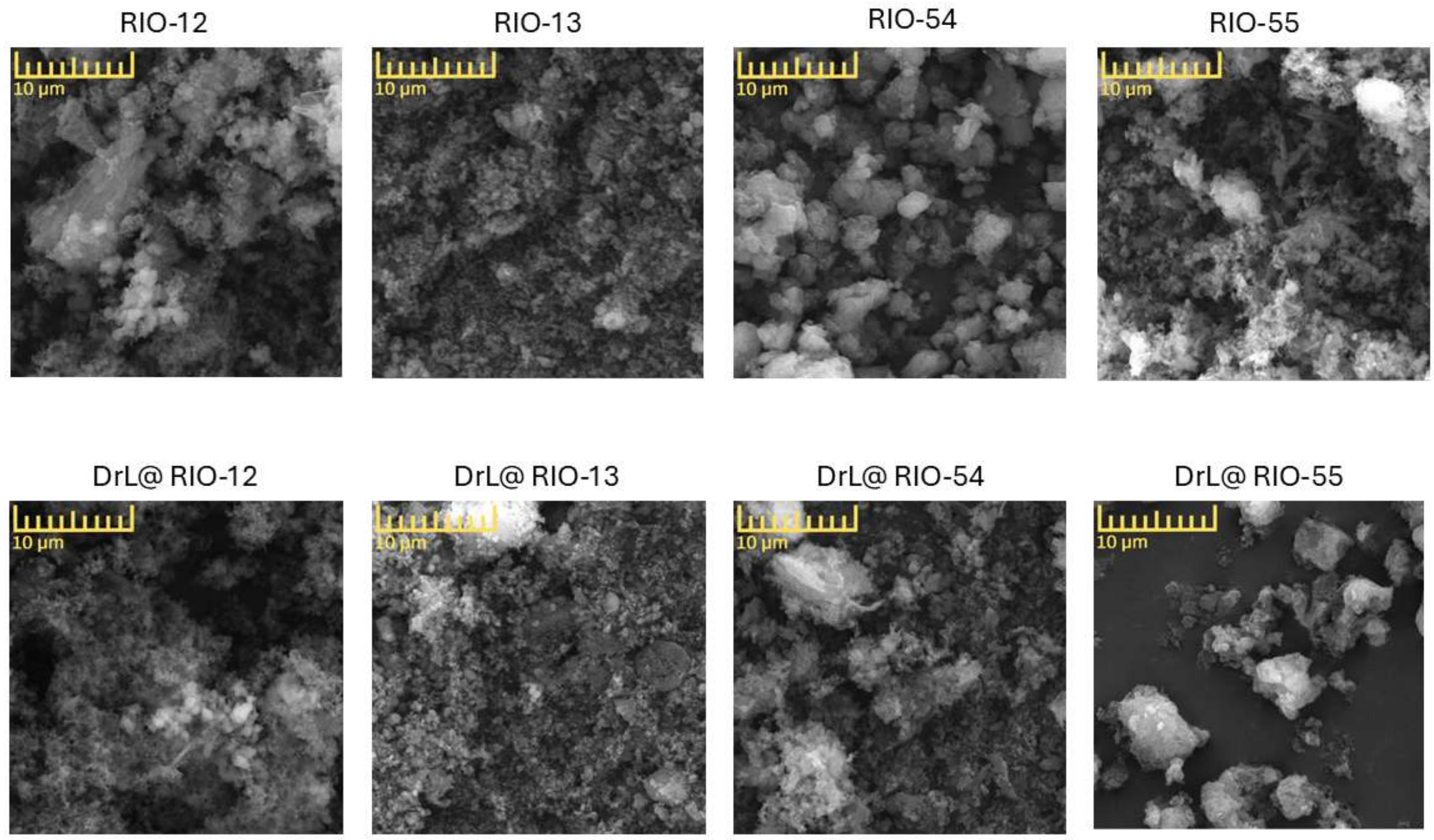

The immobilization materials with and without DrL were scaled up to 5 times and sent to Scanning electron microscopy (SEM). Simplifying, 50mg of COF were immobilized with relative mass of DrL for 17ºC, 180 rpm, 19 hours. The supernatant was led to Bradford assay, and the pellet dried by freezing drying. Scanning electron microscopy (SEM) was employed to evaluate the morphology of samples using the TESCAN VEGA3 model, operating at 15 kV. The chemical composition of samples was appraised through energy dispersive spectroscopy (EDS), which was integrated with SEM using the Bruker Nano XFlash Detector 6-10.

2. Substrate absorption at the COF

To address substrate adsorption experiments on bare COFs, we performed the catalysis, as explained previously, except by the presence of the enzyme and measured the amount of MUFHEPT that remained at the supernatant. Then, we subtracted this amount from total MUFHEPT to achieve the percentage of MUFHEPT adsorbed (FigureS5).

Figure S2: Substrate absorption at the COF

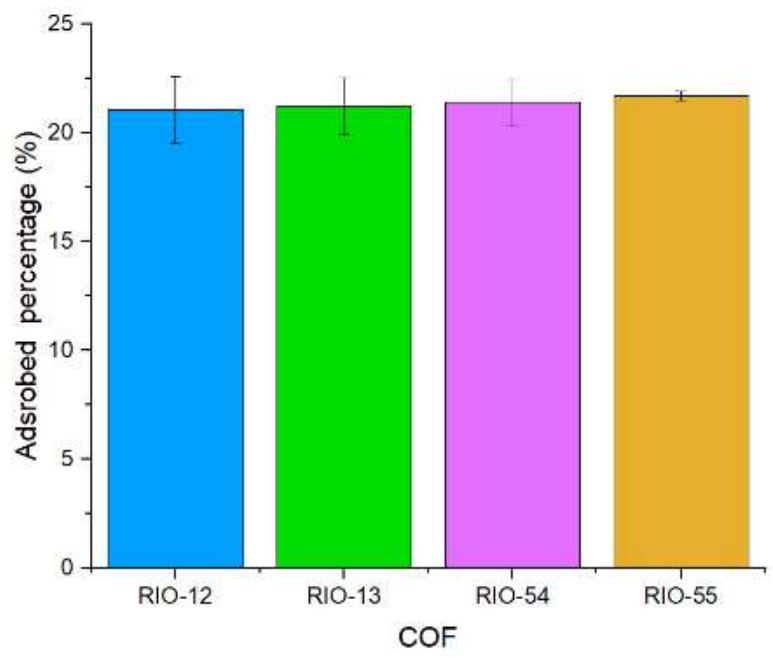
Adsrobed percentage (%)
0
5
10
15
20
25
RIO-12
RIO-13
RIO-54
RIO-55
COF
